# Space and Time Coherent Mapping for Subcellular Resolution of Imaging Mass Spectrometry

**DOI:** 10.3390/cells11213382

**Published:** 2022-10-26

**Authors:** Jun Aoki, Masako Isokawa, Michisato Toyoda

**Affiliations:** 1 Center for Biosystems Dynamics Research, RIKEN, Saitama 351-0198, Japan; 2 Graduate School of Frontier Biosciences, Osaka University, Osaka 565-0871, Japan; 3 Forefront Research Center, Graduate School of Science, Osaka University, Osaka 560-0043, Japan

**Keywords:** microscope-mode IMS, silver nanoparticle PALDI, Hirudo Verbana, endocannabinoid, neuron, epididymis, cluster analysis

## Abstract

Space and time coherent mapping (STCM) is a technology developed in our laboratory for improved matrix-assisted laser desorption ionization (MALDI) time of flight (TOF) imaging mass spectrometry (IMS). STCM excels in high spatial resolutions, which probe-based scanning methods cannot attain in conventional MALDI IMS. By replacing a scanning probe with a large field laser beam, focusing ion optics, and position-sensitive detectors, STCM tracks the entire flight trajectories of individual ions throughout the ionization process and visualizes the ionization site on the sample surface with a subcellular scale of precision and a substantially short acquisition time. Results obtained in thinly sectioned leech segmental ganglia and epididymis demonstrate that STCM IMS is highly suited for (1) imaging bioactive lipid messengers such as endocannabinoids and the mediators of neuronal activities in situ with spatial resolution sufficient to detail subcellular localization, (2) integrating resultant images in mass spectrometry to optically defined cell anatomy, and (3) assembling a stack of ion maps derived from mass spectra for cluster analysis. We propose that STCM IMS is the choice over a probe-based scanning mass spectrometer for high-resolution single-cell molecular imaging.

## 1. Introduction

Imaging mass spectrometry (IMS) is a technology that maps numerous metabolites and signaling molecules without using antibodies, fluorescent indicators, or genetically synthesized biomarkers. Matrix-assisted laser desorption ionization (MALDI) has been favorably used for biomolecular imaging because soft ionization chemistry can protect proteins and metabolites from being fragmented.

Conventional MALDI IMS uses a small laser beam as a probe and scans the sample surface sequentially while conducting mass measurements at each scanned spot. Images are generated at the end of the scan by selecting scanned spots that contain the ion of interest one by one in order to draw a final distribution pattern of the given ion image. The spatial resolution of the image depends on the probe size and sampling distance, and it typically ranges from several to over tens of micrometers, which is often too coarse for cellular levels of imaging. To circumvent the limitation in spatial resolution, subcellular organelles such as lysosomes and dense core vesicles were isolated and laid out densely in vitro for spot-targeted IMS [1,2]. Although the strategy helped image subcellular organelles, it cannot be used for high-resolution imaging in samples with complex multi-cellular organizations typically found in the human brain, even if software tools are in place for the automated processing of huge datasets.

Microscope-mode projection-type IMS acquires data differently from scanning-mode IMS. It does not scan the surface of the specimen using a small scanning probe. Instead, a wide laser beam irradiates the surface of the specimen the same way a microscope light source does. The distribution pattern of a given ion on the specimen is acquired at the time of ionization altogether simultaneously and maintained throughout the flight path of the ion. The distribution of the ion is focused by the Einzel lens and projected onto a position-sensitive ion detector. The focusing optics for ions are equivalent to the optics equipped in a microscope. The technology was initially introduced as stigmatic MALDI TOF IMS [3]. In the early prototype of the stigmatic IMS, a spatial resolution was about ten micrometers, and the mass resolution was limited to m/Δm∼500.

A major technological breakthrough for microscope-mode projection-type IMS came in 2011 when Aoki and colleagues developed a new ion extraction method called post-extraction differential acceleration (PEDA) [4]. PEDA increased mass resolutions ten times higher than previously reported and improved the spatial resolution to a sub-micrometer range independent of the laser diameter. These specifications were satisfactory for single-cell analysis and succeeded in space and time coherent mapping imaging mass spectrometry (STCM IMS).

STCM IMS utilizes large field lasers for ionization and position-sensitive detectors for imaging. The time required for the measurement is substantially short, even if the technique is applied to a large sample size. Ionization mechanisms in STCM IMS are the same as those used in conventional IMS. STCM IMS conducts standard MALDI and PALDI (nanoparticle-assisted laser desorption ionization). However, STCM IMS differs from conventional IMS in the ion optics that control the flight of ionized molecules. As the instrumentation and underlying physics have already been reported in detail elsewhere [5], here we focus on (1) basic procedures for applying STCM IMS technology to biological samples, (2) how the positional information of a given ion is maintained throughout the ion flight path, and (3) how the distribution pattern of a given ion is projected onto position-sensitive ion detectors. We will show the subcellular resolution of images for putative endocannabinoids and mediators of neuronal activities. Ion maps derived from mass spectra were applied to cluster analysis to identify molecular colocalization.

## 2. Methods

### 2.1. Animals

We used medical leeches (Hirudo Verbana) as an animal model because they have a chain of large ganglia. Individual neurons are visible discretely with an average somal diameter of 50 µm. Male reproductive organs including a pair of large epididymis can also be observed easily. These anatomical features are suited in the present study to demonstrate our newly developed technology of STCM IMS for high-resolution molecular imaging.

Leeches (4 g in weight) were purchased from Biopharm (Swansea, South Wales, UK). Leeches were kept at room temperature in a transparent glass tank filled with RO (reverse osmosis) water and 0.05% aquarium salt and constantly aerated. Bovine blood was purchased from a commercial supplier (Tokyo Shibaura Zouki, Tokyo, Japan). Leeches were fed warmed blood within a few weeks following the purchase and every three months after the initial feeding. They gained volume right after the first feed and grew 2–3 times larger in length and weight in a month.

### 2.2. Preparation of Leeches for Imaging Mass Spectrometry

Leeches were pinned ventral side down on a silicon bed and bathed in ice-cold leech ringer (in mM: 140 NaCl, 3 KCl, 1.8 CaCl${}_{2}$, 1.5 MgCl${}_{2}$, 10 glucose, 10 Hepes; pH adjusted to 7.2 with NaOH). The anterior 1/3 of the leech body, which included the male sex organs and 4th to 8th segmental ganglia, was cut and frozen immediately in hexane at −80 ${}^{\circ }$C. The remaining body part was cut and frozen at −80 ${}^{\circ }$C. In some cases, an incision was made longitudinally along the midline on the dorsal surface, and a chain of segmental ganglia (from 10th to 18th) was isolated. They were immersed in carboxymethyl cellulose (CMC) and frozen at −80 ${}^{\circ }$C. In some experiments, segmental ganglia were incubated in the leech ringer, whose potassium concentration was elevated from 3 mM to 30 mM for 15–30 min.

The frozen leech body and ganglia were mounted on a cryostat stage and sectioned at −20 ${}^{\circ }$C from the ventral side in 8 µm thick (CM3050S, Leica, Wetzlar, Germany). The sections were carefully placed on a stainless-steel metal plate at −20 ${}^{\circ }$C and dried in a vacuum for imaging mass spectrometry. Neighboring sections were mounted on a gelatin-coated glass slide for hematoxylin-eosin staining and photographed using an optical microscope BZ-X810 (Keyence, Osaka, Japan).

### 2.3. Nano-Particle Coating

Silver (Ag) nanoparticles were spattered on the surface of the sections mounted on the stainless-steel metal plate using a vacuum deposition device (MC1000, Hitachi High-tech, Tokyo, Japan) for PALDI MS (particle-assisted laser desorption ionization mass spectrometry). A discharge current of 30 mA was applied for 8 s, which we found excellent in satisfactorily coating the surface of specimens with silver nanoparticles. We were careful not to cover the surface with silver tightly, as it is necessary to create adequate spacing among individual nanoparticles [6]. Depositing the surface of the specimen with silver nanoparticles improved ionization efficiency.

## 3. Results and Discussion

### 3.1. Instrumentation of STCM IMS

**Figure 1:**
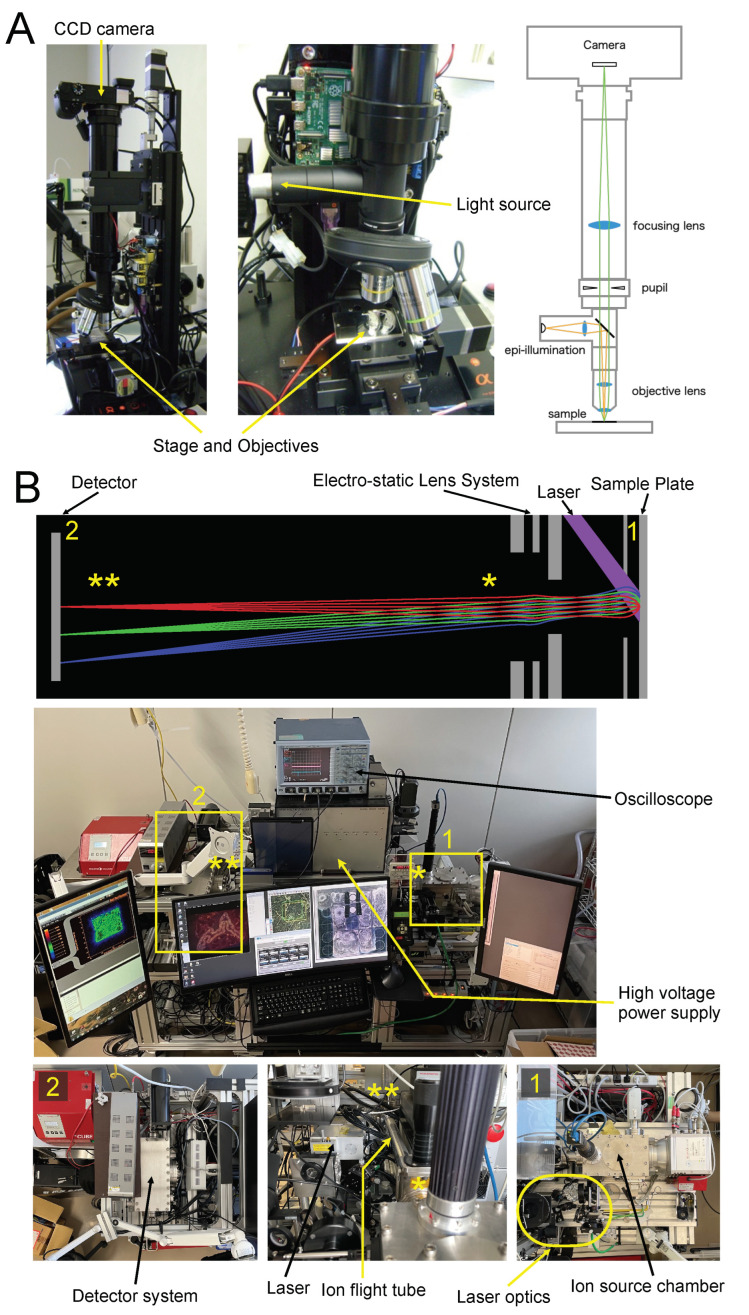
Optical microscope system and STCM IMS. (**A**) Bright-field epi-illumination microscope and CCD imaging system (ILCE-6000, Sony, Tokyo, Japan) for viewing unstained specimens (8 µm thick) mounted on a metal plate with magnifications of 10× and 20× (PLN10X and PLN20X, Olympus, Tokyo, Japan). Lens assemblies and the light path are shown on the right. (**B**) Equipment for STCM IMS. The laser path and the flight trajectories of ions are depicted schematically on top. The whole view of the instrument is photographed in the middle. The sample plate is placed inside the ion source chamber shown in Box 1. The detail of Box 1 is photographed in Picture #1 (**bottom right**). The electrostatic lens system is a part of the optics for the ion extraction system and is furnished in the same section in Box 1. The flight tube and high voltage power supply are photographed in the whole view picture and detailed in the photograph at the bottom center. Dissociated ions fly inside a meter-long ion flight tube from one end (one yellow asterisk) to the opposite end (two yellow asterisks). The detector system is enclosed in Box 2 and presented in detail in Picture #2 at the **bottom left**.

The first step in STCM IMS is to acquire optical images from 8 µm thick sections mounted on a sample plate, which was made of mirror-polished stainless steel. The optical images should be acquired before Ag spattering. We crafted a special microscope for this purpose (Figure 1A). With this scope, the entire image can be observed in a single field of view or several partial views depending on the magnification. Partial views are tiled into a composite picture for the entire image using a programmable step-motorized two-axial stage (PKP223D15A, Oriental Motor, Tokyo, Japan). Microscope images are used to integrate cell anatomy with mass spectrometric images.

After coating the sample surface with Ag nanoparticles, the sample plate was placed in the ion source chamber shown in Box 1 in Figure 1B. An ultraviolet pulsed laser (355 nm wavelength) using the third-harmonic generation of Nd: YAG (NL202, Ekspla, Vilnius, Lithuania) irradiated the section on the sample plate for laser desorption and ionization purposes. The unique features of our ion optics and trajectories are depicted in the top panel of Figure 1B.

In our irradiation system, the circular Gaussian profile of our laser beam is transformed into a rectangular uniform profile using laser beam modification optics that consist of a square core optical fiber, a rotating diffuser, and several lenses (Box 1 in Figure 1B, for details, refer to [5]). This configuration yields a homogeneously irradiated field with a width of 700 µm and a height of 690 µm. The typical measurement time is 300 s per field. Any number of irradiated fields can be measured automatically using a motorized 2-axial stage (LPS-45, PI miCos GmbH, Eschbach, Germany). For example, it takes about 1 h to finish the measurement for a total of 12 irradiated fields with a spatial resolution of one micrometer.

Molecules ionized from the sample surface are extracted by the electric field generated by the extraction electrode shown in Figure 1B. The extracted ions are emitted to the ion flight tube where PEDA is equipped to increase a mass resolution [4]. Einzel lenses, a type of electrostatic lens, collect emitted ions, focus ion images, and project them onto a delay line detector (DLD) with magnification equivalent to 20× to 50×. DLD is a time- and position-sensitive detector with a spatial resolution of 50 µm (DLD40, RoentDek Handels GmbH, Kelkheim, Germany). It measures the flight time and the space-wise distribution of projected ions at the same time simultaneously. The trajectories of emitted ions are maintained accurately throughout the flight with the ion optics designed by computer simulation not to lose the initial ionization site on the sample surface, which is explained schematically by the illustration and picture panels in Figure 1B.

### 3.2. Ion Flight Mode and Spatial Resolution in STCM IMS

**Figure 2:**
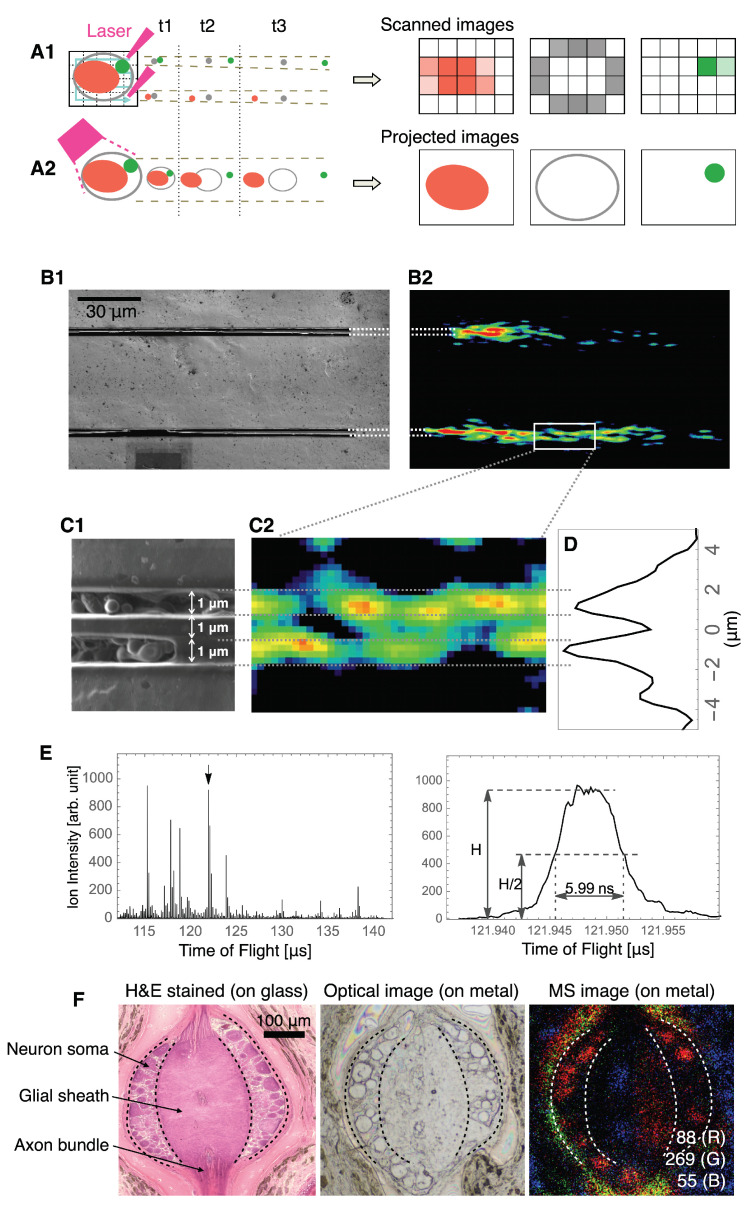
Ion flight mode and spatial/mass resolution in STCM IMS. (**A**) Schematic diagrams for the flight mode of ions in STCM IMS and the conventional scanning-type IMS. (**A1**) depicts the ion flight mode for conventional scanning-type IMS and (**A2**) depicts the ion flight mode for STCM IMS. Red, green, and gray spots indicate the distribution patterns of three ionized molecules that possess different masses. Distances among different color spots in t1, t2, and t3 indicate differences in flight times based on the unique mass value assigned in each ionized molecule. On the right, three images created by the conventional scanning-type IMS (**upper three**) and by STCM IMS (**lower three**) are shown. (**B**) Electron microscope images of physicochemical test lines for spatial resolution. Two 1 µm wide metal slits, each painted with crystal violet, were placed 1 µm apart (**B1**). Ions extracted from crystal violet were visualized as two separate lines (**B2**). (**C**) Enlarged view of the test lines (**C1**) and magnified MS signals (**C2**) taken from the portion enclosed by the yellow box in B2. (**D**) Two peaks in the intensity curve quantitatively confirm the graphical presentation of MS signals in (**C2**). (**E**) Evaluation of mass resolution using the peak identified as the crystal violet ion at 121.948 µs in the mass spectra. (**F**) The leech somatic ganglion sectioned at 8 µm thick and stained with hematoxylin and eosin (**left**). An unstained section of the ganglion with the same thickness (**middle**), mounted on a mirror-polished stainless-steel plate, is viewed by our special microscope shown in Figure 1A. Subcellular localization of three ionized molecules, *m*/*z* 88 (R), *m*/*z* 269 (G), *m*/*z* 55 (B), imaged in the same section are shown in the middle panel (**right**). Dashed lines in each panel indicate anatomical boundaries. A calibration bar on the left panel is 100 µm and is shared by all three panels.

STCM IMS differs from conventional scanning IMS in the ion flight mode (Figure 2A). In conventional scanning IMS, emitted ions are located by a scanning probe. Hence, the spatial resolution of the ion image is determined by the sampling distance of the scan and limited to the size of the scanning laser beam (Figure 2(A1)). In contrast, STCM IMS replaces a scanning laser probe with a large field laser beam. This configuration allows a given ion to be directly projected to the detector with a sub-micron of spatial resolution independent of the size of the laser beam (Figure 2(A2)).

Physicochemical tests were conducted to assess the spatial resolution of STCM and presented together with the test results in Figure 2B–D. Two 1 µm wide slits are placed 1 µm apart and imaged by STCM for the presence of a crystal violet molecule in each slit. The test result revealed that the crystal violet molecule was ionized and visualized in two separate lines, which suggested that STCM has a sub-micron order of spatial resolution for imaging.

The mass resolution was evaluated from the peak identified as the crystal violet ion at 121.948 µs shown in Figure 2E. The width of the half height of the peak was measured as 5.99 ns. Thus, the mass resolution was calculated as m/Δm=t/2Δt∼10,162.5 by definition.

We next show a result of imaging in biological samples obtained by STCM. The anatomy of the leech segmental ganglion is presented with cell bodies of neurons and central glial sheaths through which axons project longitudinally (Figure 2F). The left panel shows an 8 µm thick section stained with hematoxylin and eosin, whereas the middle panel shows an unstained section with the same thickness, mounted on a mirror-polished stainless-steel plate and viewed by our special microscope shown in Figure 1A. In both panels, individual neurons are visible, and the preparations are considered sufficient to be used for single-cell-level analysis. The right panel shows an example of mass spectrometric images obtained by STCM from the same section shown in the middle panel. STCM visualized numerous sub-cellular localizations of three ionized molecules, *m*/*z* 58, *m*/*z* 88, and *m*/*z* 269, in multiple neurons. Although we did not characterize the molecular nature of these ions, the result suggested that the specification tested for STCM in Figure 2B–E is sufficient to detect multiple ions in biological samples and image them on a subcellular scale of spatial resolution.

Biological samples contain numerous molecules such as proteins, metabolites, lipids, and nucleic acids. In addition, biological samples are structurally more complex than a monolithic layer of crystal violet placed on the stainless-steel plate. We often experience that biological samples are challenging to be imaged at a sub-micron scale of spatial resolution. Careful preparation of samples and the adequate selection of ionization strategies are critically important not to lose the demonstrated quality of ionization efficiency and image resolution provided by STCM. The surface condition of samples may be altered as a result of experimental manipulations or possible pathologies. Such factors could impose restrictions and hamper us from achieving a theoretically defined performance.

We used standard calibration methods of the third-order polynomial fitting with mass spectral peaks of *m*/*z* 23 (${}^{23}$Na), *m*/*z* 39 (${}^{39}$K), *m*/*z* 107 (${}^{107}$Ag), and *m*/*z* 109 (${}^{109}$Ag). The reason for using these ions for calibration is that Na and K are naturally abundant in biological specimens and provide strong signals stably. We also use silver particles routinely to improve ionization efficiency (see Section 2).

Data obtained with STCM IMS can be exported to BioMap (Novartis, freeware) for spectrum-based image-viewing and identifying molecules based on their *m*/*z* values. Data can also be exported to Image-J (NIH, freeware) for image arithmetic, color merge, overlay, segmentation, and selecting the region of interest (ROI).

### 3.3. Low Molecular Mass Organic Compounds Imaged in the Epididymis

Micromolecules with a size on the order of 1 nm are low molecular weight (<*m*/*z* 1000) organic compounds that may regulate a biological process. We targeted molecules with this range of ionized mass (*m*/*z*) and conducted STCM IMS in the horizontally sectioned leech body in situ.

**Figure 3:**
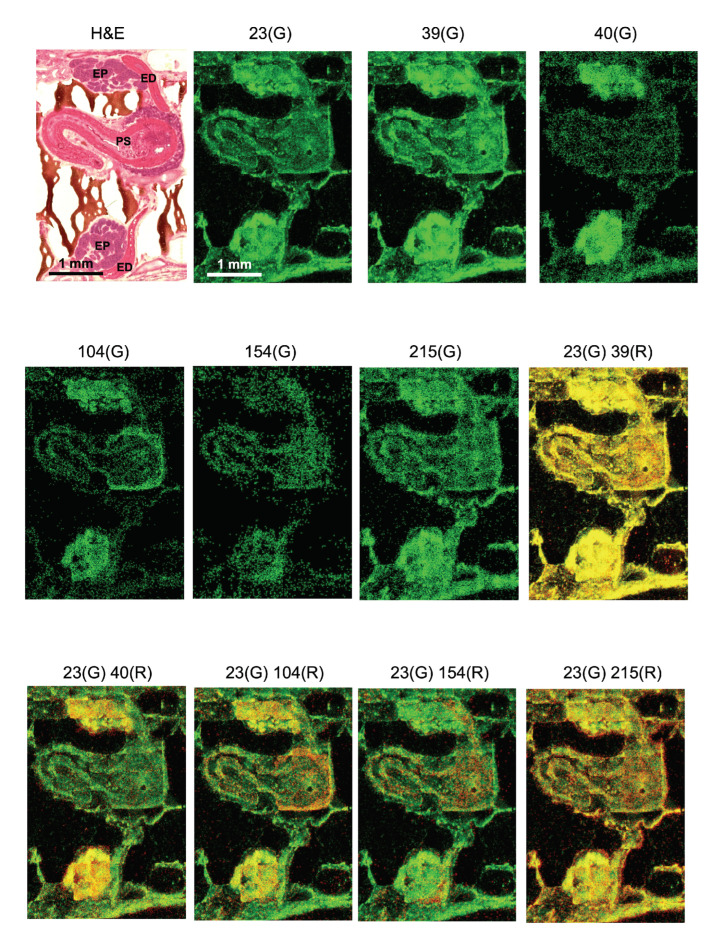
Low molecular mass organic compounds imaged in the epididymis by STCM IMS. The morphology of the male reproductive system is shown at the left top with H&E staining (ED: ejaculatory duct; EP: epididymis; PS: penis sheath; calibration: 1 mm). Localization of ionized sodium (Na, *m*/*z* 23), ionized potassium (K, *m*/*z* 39), and ionized calcium (Ca, *m*/*z* 40) are shown in green. In addition, *m*/*z* 104, *m*/*z* 154, and *m*/*z* 215 are imaged in green (see text for the assignment of these ions). The overlay of sodium ions in green (G) and five other ionized molecules in red (R) accentuated differences in the ion distribution pattern and helped us to identify the localization pattern unique to individual molecules. Calibration (1 mm) in 23G is shared by the rest of the MS images.

Dense MS signals were detected in the epididymis, as shown in Figure 3. The epididymis is a male reproductive organ comprising a heavily convoluted single tube. It collects immature sperm. Sperms mature and acquire their fertilizing capacity while migrating inside the duct of the epididymis [7]. Leeches have a pair of epididymis situated symmetrically along the midline near the fifth sex ganglion (Figure 3A).

Basic ions that compose biological specimens such as sodium ions (Na, *m*/*z* 23), potassium ions (K, *m*/*z* 39), and calcium ions (Ca, *m*/*z* 40) are presented as MS images individually (Figure 3). Na and K showed a similar pattern of distribution in the epididymis and the penis. In contrast, Ca showed strong signals in the epididymis but almost no signals in the penis suggesting differing roles of these ions in male reproductive organs. In addition, *m*/*z* 104, *m*/*z* 154, and *m*/*z* 215 exhibited discrete patterns of localization that were uniquely different from each other in the epididymis.

We did not conduct on-tissue chemical derivatization for increased ionization efficacy or use external references and internal standards for biochemical confirmation. We did not consider inorganic compounds and synthetic chemicals in interpreting our results. They were excluded as unlikely candidates in characterizing a given *m*/*z*. On the other hand, biological molecules such as neurotransmitters were preferentially considered strong candidates in characterizing imaged products. We also referred our data to anatomical and immunohistochemical findings reported by others. MS/MS cannot be conducted by STCM IMS because it hampers the maintenance of the mass images generated by the STCM. Finally, the mass resolution reported in the leech specimen was less than the calculated resolution reported in Figure 2E. This is because the acquisition of high mass resolution tends to distort ion images, particularly around the periphery. We prioritized the quality of the image over mass resolution.

The ionized molecule with *m*/*z* 104 was found to be localized in the epididymis and the atrium of the penis sheath (Figure 3). Specifically, the epithelial plexus attached to the outer edge of the atrium strongly exhibited *m*/*z* 104. Choline ([M]^+^), GABA ([M + H]^+^), and serine ([M − H]^−^) share the identical *m*/*z* 104. As STCM IMS was conducted with a positive mode, the possibility of *m*/*z* 104 as serine is unlikely.

In the literature, it was reported that GABA was present in the vascular system [8] and the plexus at the atrium where intricately developed networks of blood vessels occupy. The presence of GABA in the head (caput) of the epididymis was reported with a concentration 10–50 times lower than in the brain [9,10]. Thus, our result of imaging *m*/*z* 104 with STCM IMS may have captured putative GABA signals in the male reproductive system and provided anatomical information regarding the localization of GABA in the epithelial plexus attached to the outer edge of the atrium of the penis sheath. GABA was also reported to be a major neurotransmitter in leeches [11]. Together, available evidence suggests that GABA not only regulates sperm kinematics and functions in the epididymal duct but also controls peristalsis of epididymal muscles through active GABAergic terminals that are widespread over the myocytes of the epididymis. Further examination is required to determine the molecular nature of currently observed *m*/*z* 104.

The ionized molecule with *m*/*z* 154 was observed in the bulb of the epididymis bilaterally and in the penis wall where musculature dominated (Figure 3). Leucine ([M + Na]${}^{+}$), octopamine ([M + H]${}^{+}$), and dopamine ([M + H]${}^{+}$) share an identical *m*/*z* 154. Yet, the localization of *m*/*z* 154 in the muscle-rich portion of the male reproductive organ suggested us the possibility of characterizing *m*/*z* 154 as putative dopamine (DA). Indeed, there is supportive evidence that the concentration of DA was 60–70 ng/g of tissue in the rat epididymis [12]. Individual *m*/*z* 154 signals might reflect DA-containing motor neuron axons terminated on the epididymis muscles.

When the image of *m*/*z* 154 was compared with the image of *m*/*z* 104, distribution patterns were different, with some portions overlapping and other portions mutually exclusive. Since DA was reported to stimulate chloride transport through epididymal epithelia [13], and GABA is known to regulate the chloride concentration gradient across the plasma membrane, it is of interest to further elucidate a physical distance between *m*/*z* 104 ions and *m*/*z* 154 ions. We expect that such an attempt could advance our understanding of how these two ionized molecules regulate a site-specific function in local cells of the epididymis.

The ionized molecule with *m*/*z* 215 exhibited higher MS signals in the epididymis than in the penis (Figure 3). The regional localization of *m*/*z* 215 was specific and differed from the localization pattern of either *m*/*z* 104 or *m*/*z* 154. We found only serotonin (5-HT, [M + K]${}^{+}$) to be a likely molecular candidate for *m*/*z* 215. There is an interesting report in the leech that 5-HT immunoreactivity was observed intensely in the epididymis and mildly in the penis [14]. The rat male reproductive organs were immunoreactive to 5-HT [15,16], and the concentration of 5-HT was around 25–30 ng/g of tissue in the mammalian epididymis [13]. Together, the above evidence suggests that STCM IMS likely confirmed the localization of putative 5-HT in the leech epididymis.

Finally, the color overlay of *m*/*z* 104, *m*/*z* 154, and *m*/*z* 215 with Na (*m*/*z* 23) accentuated the unique distribution pattern of each molecule. It helped us identify mutually exclusive or inclusive localization of these molecules in the epididymis. We tentatively assigned three possible neurotransmitters to these ionized molecules. There are reports by others regarding the MS imaging for neurotransmitters (Sugiyama et al., 2019; with MALDI [17]), MS imaging in the epididymis (Lagarrigue et al., 2020; with MALDI [18]), and MS imaging in the leech (Meriaux et al., 2011; with MALDI and SIMS [19]). However, we did not find any MSI data that reported neurotransmitters in the epididymis of the leech. We think it is technically advantageous to use STCM IMS in the event of measuring multiple molecules simultaneously from the same biological sample and visualizing them in anatomically identifiable images. When more than several molecules are involved and the intended imaging includes bioactive lipids such as endocannabinoids, our STCM technology would be the best choice since the visualization of bioactive lipids cannot be easily accomplished by antibody-based fluorescent microscopy.

### 3.4. Endocannabinoid 2-AG Imaging in Neurons

**Figure 4:**
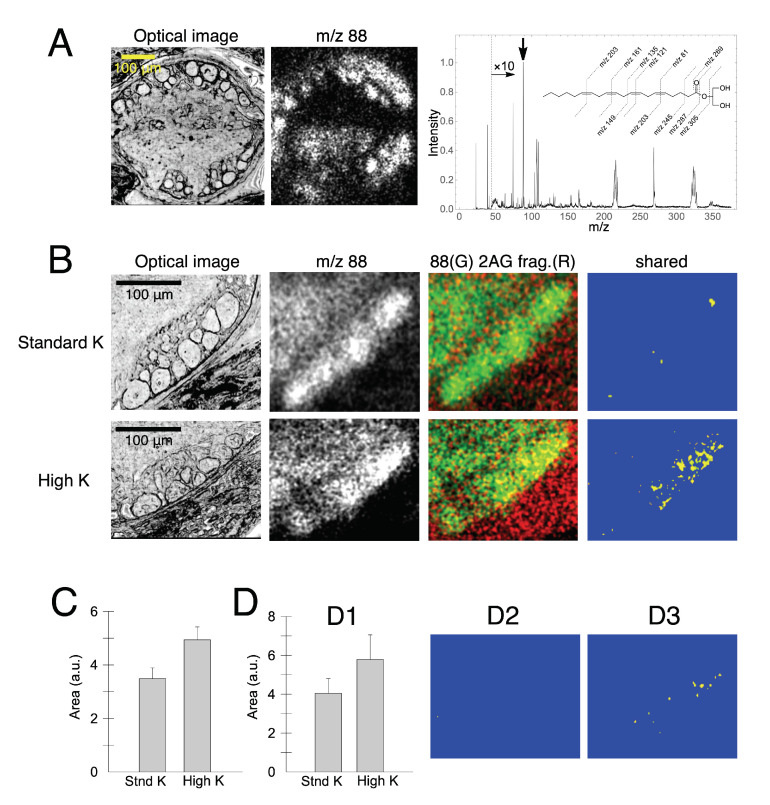
Neuronal localization of putative 2-AG. (**A**) Unstained optical image of a somatic ganglion (**left**) and an image of *m*/*z* 88 (**right**). *m*/*z* 88 is pointed by an arrow in the mass spectrum. Fragments of 2AG are depicted above the spectrum. (**B**) Images of 2-AGfragSum (red) and *m*/*z* 88 (green) in a standard concentration (3 mM) of potassium (K) (in the upper set of data) and those in a high concentration (30 mM) of K (in the lower set of data). Yellow segments represent the colocalization of 2-AGfragSum (red) and *m*/*z* 88 (green). (**C**) A summary graph indicating an increased localization of putative 2-AG in high K-treated neurons (student *t*-test, *t* = 2.358, *p* = 0.027, *N* = 12 ganglia in each experiment). (**D**) Colocalization of *m*/*z* 40 (likely calcium) and *m*/*z* 88. (**D1**) A subtle increase of *m*/*z* 40 in high K-treated neurons (student *t*-test, *t* = 1.157, *p* = 0.274, *N* = 6 in each experiment). (**D2**,**D3**) Yellow ROIs indicated calcium images in neurons treated with a standard K vs. high K, respectively.

Endocannabinoid 2-arachidonoylglycerol (2-AG) is a representative bioactive lipid messenger. 2-AG forms a proton adduct ([M + H]${}^{+}$, *m*/*z* 379) in a positive mode. In addition, the sodium adduct ([M + Na]${}^{+}$, *m*/*z* 401), potassium adduct ([M + K]${}^{+}$, *m*/*z* 417), and ammonium adduct ([M + NH${}_{4}$]${}^{+}$, *m*/*z* 396) can be measured. 2-AG also forms adducts with silver ions ([M + ${}^{107}$Ag]${}^{+}$, *m*/*z* 485; [M + ${}^{109}$Ag]${}^{+}$, *m*/*z* 487) [20]. Regarding the fragmentation of 2-AG, a total of 11 fragments were reported [21] (*m*/*z* 81, 121, 135, 149, 161, 203, 245, 269, 287,305, 361) (inset in the spectrum in Figure 4A). In addition, there are two isomers, 2-AG and 1-AG. Although 1-AG is not biologically active, 1-AG and 2-AG share the same *m*/*z* and cannot be separated by MALDI TOF IMS. Methanol and ethanol are particularly effective in inducing isomerization [21]. These solvents were avoided in the present study of STCM IMS.

We imaged a depolarization-induced release of 2-AG in the leech neuron. The production and mobilization of 2-AG upon neuronal depolarization is a widely reported phenomenon across many species, including rodents [22] and leeches [23]. 2-AG is produced from diacylglycerol upon depolarization by diacylglycerol lipase and degraded by monoacylglycerol lipase. The synthesis of 2-AG was reported to occur within a second after depolarization, and the degradation of 2-AG was reported to begin after the synthesis in several seconds [22]. Conditional production and short lives of neuronal 2-AG make it challenging to conduct successful IMS.

To maximize the chance of capturing every possible trace of 2-AG in neurons, we generated a composite image of 2-AG by summing all adducts and fragments. Putative 2-AG was imaged as 2-AGaddctSum and 2-AGfragSum. In parallel with 2-AG imaging, a molecule with *m*/*z* 88 was imaged as a neuronal marker, as it showed an excellent correspondence with neuron morphology in the leech ganglion (Figure 4A). However, we could not characterize the molecular identity of *m*/*z* 88 (see MS spectrum in Figure 4A).

We induced neuronal depolarization by applying a high concentration of potassium (K). It was assumed that a release of 2-AG occurred from neurons when the image of 2-AGfragSum overlapped with the image of *m*/*z* 88. The overlapped area between 2-AGfragSum (red) and *m*/*z* 88 (green) was exhibited as yellow. The yellow section was selected as a region of interest (ROI) against a blue background for better visual assessment (Figure 4B). When yellow ROIs were compared between the ganglia treated with 3 mM K (upper) and those treated with 30 mM K (lower), the total area of yellow ROI was greater in the ganglia treated with the higher concentration of potassium (K) (Figure 4C). The amount of 2-AG produced by neuronal depolarization was reported to be around 4.0 nmol/g of tissue in the rat hippocampus [24]. In the present study, STCM detected 2-AG-producing neurons individually and imaged them with subcellular spatial resolution. Future experiments should reveal cellular patterns of 2-AG production and release paths among local neurons in response to various forms of depolarization.

Finally, depolarization was reported to induce an increase in cytosolic calcium to around 4 µM [25]. However, we did not confirm any calcium increase in the image of *m*/*z* 40 in the present study (Figure 4D). We suspect that a calcium increase probably occurred upon depolarization but was not measured successfully as an increase of *m*/*z* 40 by STCM IMS. An additional technique may be required to track the time course of calcium transients to supplement a technical limitation in imaging mass spectrometry.

### 3.5. Endocannabinoid Imaging and Cluster Analysis in the Epididymis

The epididymis contains various molecules in addition to neurotransmitters, and many of them show differing concentrations between the head (caput) and the tail (cauda) of the epididymis [26]. Differences in molecular contents within the epididymis are explained to help regulate the maturation of sperms [27]. We think it is important to investigate diverse distributions of identified and unidentified molecules as much as possible to better understand physiological functions and disease states in the epididymis.

Endocannabinoid 2-AG is a molecule that shows a differing concentration within the epididymis, having a twice higher concentration in the head than in the tail [28]. In mammals, 2-AG is produced in the duct of the epididymis [29] and the testis [30] and is known to inhibit sperm motility [29]. However, there is no report for cellular localization of 2-AG in the leech epididymis. Serotonin (5-HT) [31] and the cognate receptors (5-HT2 and 5-HT1b) are involved in the production of 2-AG [32], and immunohistochemical studies showed that serotonin (5-HT) and 5-HT receptors were expressed in the leech epididymis [14,32]. We imaged *m*/*z* 215 and interpreted it putative 5-HT in the leech epididymis in the present study (see Figure 3). The accumulating evidence led us to investigate a local production of homeostatic 2-AG in the epididymis with STCM IMS. We adopted the identical method used for 2-AG imaging in neurons.

**Figure 5:**
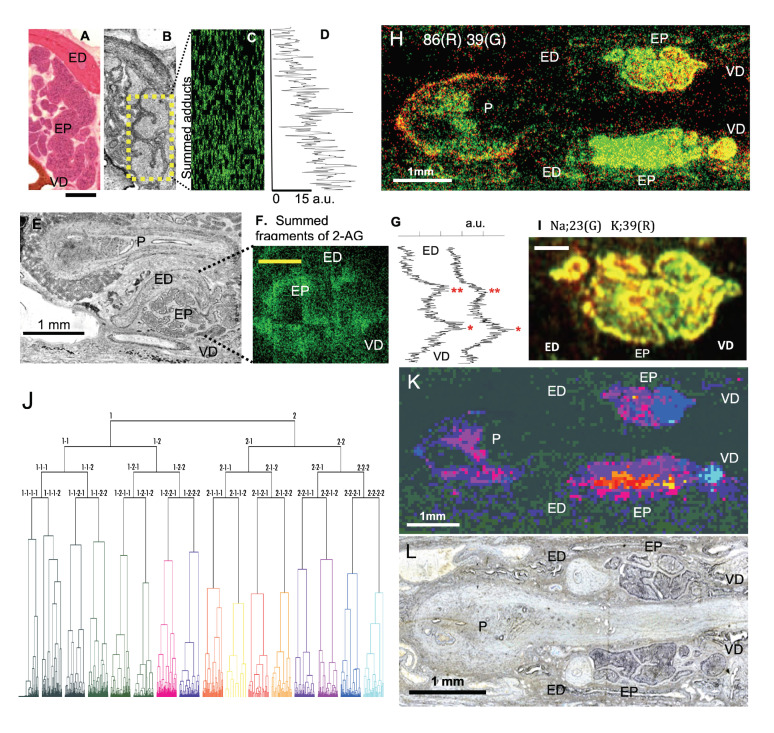
(**A**) An epididymis section stained with hematoxylin-eosin (8 µm thick). (**B**) An optical image taken from a neighboring section in the same epididymis shown in (**A**). (**C**) The image of 2-AGaddctSum acquired in the same section shown in (**B**). The ion image was taken from the area enclosed by the yellow box. (**D**) A plot for the average signal intensity calculated in individual pixel rows in 2-AGaddctSum shown in (**C**). The plot exhibited that the signal was 15 times stronger in arbitrary units in the bottom pixel row (near VD close to the head) than in the top pixel row (near ED close to the tail), suggesting a gradual decrease in 2-AG signals from the head to the tail. (**E**) An optical image of an epididymis section. (**F**) The image of 2-AGfragSum acquired in the same section in (**E**). (**G**) A plot for the average signal intensity in individual rows in 2-AGfragSum. The peak intensity was higher in the head (one asterisk) than the tail (two asterisks) of the epididymis. (**H**) Image for *m*/*z* 86. (**I**) Distribution of Na (*m*/*z* 23) and K (*m*/*z* 39) were color-coded in the epididymis. Na (green) was higher in the head of the epididymis, whereas K (red) was higher in the tail of the epididymis. (**J**) Individual segments (defined in the text) were organized into hierarchical clusters (dendrogram) based on the similarity in the peak profile of the mass spectrum. Segments were categorized into 16 groups at the fourth branching point in the dendrogram. Each cluster is marked by a different color. (**K**) The STCM image was colored according to the color code in the dendrogram in (**J**). (**L**) The optical microscope image that corresponds to the MS image shown in (**H**,**K**). EP: epididymis; ED: ejaculatory duct; P: penis; VD: vas deference. Calibrations: 300 µm (**A**,**F**,**I**).

In 2-AGaddctSum, putative 2-AG showed strong signals in the head of the epididymis near the vas deference (VD), but the signal intensity gradually decreased in the tail near ED (Figure 5A–D). In 2-AGfragSum, putative 2-AG showed two peaks in the intensity curve. The higher concentration corresponded to the head of the epididymis (Figure 5E–G). The result agreed with a previous report that a twice higher concentration of 2-AG was measured in the head (9000 pmol/g of tissue) than in the tail (4500 pmol/g of tissue) of the epididymis [30]. Our data suggest that STCM IMS can image naturally existing homeostatic 2-AG from a highly convoluted duct of the epididymis in situ in reference to the anatomy of this organ. We are planning to address the effect of 5-HT in the production of 2-AG and the involvement of other signaling molecules in the mobilization of 2-AG in the epididymis.

In addition to 2-AG, a small molecule with *m*/*z* 86 showed a strong localized signal in the head of the epididymis near VD (Figure 5H). Although we did not characterize *m*/*z* 86, it may be a lipid fragment because phosphatidylcholines and phosphatidylethanolamines were rich in the epididymis and showed different concentrations between the head and the tail of the epididymis [18].

Sodium (Na, *m*/*z* 23) and potassium (k, *m*/*z* 39) are abundant in the epididymal cells. In the present study, Na and K were localized complementary between the head and the tail of the epididymis (Figure 5I). Potassium showed a high concentration in the tail. The result agreed with a previous report and explained that a high K assisted sperms with gaining mobility and exiting the epididymis smoothly [26].

It is interesting to discover how many molecules in total may show uneven distribution between the head and the tail of the epididymis. Cluster analysis is a powerful method [33] for finding an answer, as it can unveil the distribution of numerous identified and unidentified molecules in the epididymis.

We divided the entire image frame used for the epididymis into segments of 5 µm × 5 µm each. The mass spectrum was obtained from every segment regardless of whether a segment included the epididymis. Major peaks in the mass spectra in each segment were used as multidimensional variables to determine a mass profile of a given segment. The mass profiles in individual segments were collected and assembled into a hierarchical pyramid (dendrogram) using the Ward’s method [34]. Segments with similar mass profiles were positioned close in the dendrogram (Figure 5J). We categorized the segments into 16 groups at the fourth branching point in the dendrogram. Segments that had a strong resemblance in the mass profile were assigned to similar colors. Each point in the image was painted based on categorization by cluster analysis (Figure 5K). The colors painted in the image correspond to the colors in the dendrogram. Color-coded clusters separated molecules in various anatomical subdivisions of the epididymis (Figure 5K,L). More specifically, blue-colored segments dominated the entrance and the head portion of the epididymis. However, the color changed to red and purple in areas close to the tail of the epididymis. The color change indicates that molecular contents evolve from the head of the epididymis toward the tail of the epididymis. In the atrium of the penis, where matured sperms are stored for ejaculation, the color is almost exclusively purple, indicating little molecular diversity in this organ. In sum, the result of our cluster analysis suggested that STCM IMS comprehensively measured numerous molecules in differing concentrations from various sub-compartments of the epididymis and coherently integrated them space- and flight-time-wise into a multi-layered molecular map for massive parallel screening.

## 4. Conclusions

We discussed the merit of using STCM IMS to investigate signaling molecules in biological specimens. STCM IMS extracts and detects ionized molecules without mixing their flight trajectories inside the ion flight tube. Consequently, the technology preserves positional cues of all *m*/*z* in mass spectra with subcellular spatial resolution. It generates a two-dimensional chart of ionized molecules as an anatomically accurate image. Using STCM IMS, we imaged stimulus-induced production of endocannabinoids in single neurons of the leech segmental ganglion and homeostatic 2-AG and the mediators of neuron activities in the epididymis. Successful outcomes for imaging numerous ions with subcellular resolution promoted a comprehensive cluster analysis for investigating molecular lineage and constructing multi-layered molecular maps. Our team is developing a new time- and position-sensitive detector to accommodate multi-hit ions typically occurring in MS imaging and achieve higher spatial resolution.

## Data Availability

The raw and processed data reported in this study will be made available upon request.
